# Case report: Microangiopathic hemolytic anemia and thrombocytopenia in a child with Brucella infection

**DOI:** 10.3389/fped.2023.1139622

**Published:** 2023-06-15

**Authors:** Atheer Alkhunein, Ahmed Albraikan, Maria Alayed, Wesam Althaqafi, Musaed Alharbi

**Affiliations:** ^1^ Department of Pediatrics, King Abdullah Specialist Children Hospital, Ministry of National Guard-Health Affairs (NGHA), Riyadh, Saudi Arabia; ^2^ King Abdullah International Medical Research Centre, King Abdulaziz Medical City, Ministry of National Guard-Health Affairs (NGHA), Riyadh, Saudi Arabia; ^3^ College of Medicine, King Saud bin Abdul-Aziz University for Health Science, Ministry of National Guard-Health Affairs (NGHA), Riyadh, Saudi Arabia; ^4^ Department of Pediatric Infectious Diseases, King Abdullah Specialist Children Hospital, Ministry of National Guard Health Affairs (NGHA), Riyadh, Saudi Arabia

**Keywords:** case report, Brucella, thrombocytopenia, microangiopathic hemolytic anemia (MAHA), ADAMTS-13 (a disentegrin-like and metalloprotease with thrombospondin type 1 motif), thrombotic thrombocitopenic purpura

## Abstract

Thrombotic thrombocytopenic purpura (TTP) is a diffused microvascular occlusive disorder characterized by microangiopathic hemolytic anemia (MAHA), thrombocytopenia, and ischemic organ dysfunction. In addition, this condition has been linked to an absence or dysfunction of ADAMTS13. Although TTP can occur due to various factors, such as bacteria, viruses, autoimmune disorders, drugs, connective tissue conditions, and solid tumors, it is a rare hematological complication associated with brucellosis. We describe the first case of a 9-year-old boy with acquired TTP with undetectable ADAMTS-13 assay secondary to Brucella infection. After initiating antimicrobial therapy, symptoms and laboratory abnormalities improved dramatically, with no recurrence of TTP in subsequent follow-ups.

## Introduction

Brucellosis is the most common zoonotic disease in Saudi Arabia, with an incidence rate of 50–500 cases per 1,000,000 population (1, 2). Incidence of brucellosis among children ranges from 20% to 30% among endemic regions (2). It is a multisystem infection with broad-spectrum clinical manifestation. The most frequently involved sites are the musculoskeletal system, spleen, liver, and bone marrow (3, 4). Anemia, leukopenia, and pancytopenia are common hematological manifestations of brucellosis (4). Although there are few case reports of thrombotic microangiopathy associated with brucellosis, none have performed an ADAMTS13 (A Disintegrin and Metalloprotease with ThromboSpondian Type 1 motifs 13) test. This report presents the case of a 9-year-old boy with acquired thrombotic thrombocytopenic purpura (TTP) from brucellosis infection with an undetectable ADAMTS13 assay.

## Case report

A 9-year-old previously healthy Syrian boy was admitted with a 1-day history of epistaxis, hematemesis, hematuria, and melena associated with periumbilical abdominal pain. He also has been complaining of pallor, decreased activity and appetite, and weight loss for a month prior to his presentation. A week before his admission, there was a noticeable increase in his pallor. Upon presentation, he was hypoactive, pale, and jaundiced. He was normothermic and hemodynamically stable. System examination revealed hepatosplenomegaly small palpable lymph node in the supraclavicular and inguinal region. The remainder of his physical examination was unremarkable.

His initial Laboratory tests at admission showed a picture of bi-cytopenia in the form of anemia and thrombocytopenia. Platelet 11 × 10^9^/L (normal range: 150–400 × 10^9^/L); hemoglobin 97 mg/L (normal range: 113–150 mg/J); white blood cells 10.2 × 10^9^/L (normal range: 4–12 × 10^9^/L); and reticulocyte counts 2.45% (normal range: 0.5%–1.5%). Peripheral blood smear showed red blood cell fragmentation (Schistocytes) at 1.45%, and white blood cells, mainly neutrophilia, displayed reactive changes in forms of toxic granulation.

Lactate dehydrogenase was 1,021 U/L (normal range: 125–220 U/L); aspartate aminotransferase 46 IU/L (normal range: 5–34 U/L); alkaline phosphatase 143 IU/L (normal range: 156–369 U/L); total bilirubin 41.3 µmol/L (normal range: ∼20.5 µmol/L); and direct bilirubin 12.7 µmol/L (normal range: ∼8.6 µmol/L). Blood urea nitrogen and creatinine were within normal limits. Urinalysis showed hematuria and hemoglobinuria. Coagulation screening tests were within the normal range; fibrinogen level was decreased to 1.72 g/L (normal range: 1.5–4.1 g/L); Coombs test was negative.

Initially, the patient was diagnosed with atypical idiopathic thrombocytopenic purpura (ITP) and treated with Intravenous Immunoglobulin (IVIG) 1 g/kg. He received two doses of IVIG without response, and his Hgb and platelet counts declined further.

In order to rule out bone marrow infiltration, bone marrow aspirations (BMA) and biopsy were performed. The preliminary report of the BMA did not suggest malignancy; however, it showed megakaryocytes which is suggestive of immune destruction. Therefore the patient was started on a six days trial of pulse steroid therapy (intravenous methylprednisolone 2 mg/kg/day divided twice a day), with no improvement in Hgb or platelet count. The final result of BMA revealed peripheral destruction of blood cells, indicating microangiopathic hemolytic anemia (MAHA), likely TTP, congenital or acquired due to an immune reaction to ADAMTS-13; this diagnosis was supported by repeated peripheral blood smear showing Schistocytes fragments of red blood cells >2.5% (Normal range <1.5%), accordingly a five days trial of fresh frozen plasma transfusion was initiated, in which the patient showed improvement in LDH and platelet levels.

In addition, bone marrow biopsy results showed morphologic evidence of frequent small epithelioid non-necrotizing granuloma. Considering that this is a nonspecific finding that could be attributed to various etiologies, including infections (viral, bacterial, and tuberculosis), infiltrating diseases such as sarcoidosis, and malignancies, further investigation has been pursued. Viral surveillance, including EBV, CMV, and HIV were negative. QuantiFERON-TB Gold was negative. Abdominal ultrasound and computerized chest and abdominal tomography (CT) scan showed splenomegaly with no significant lymph node enlargement. Since Brucella is endemic in Saudi Arabia and the patient had recently consumed a significant amount of unpasteurized goat milk and cheese, brucella titers were sent, and the result showed Brucella abortus 1:5,120 (normal range: <1:160), and Brucella melitensis 1:20,480 (normal range: <1:160).

A combination of Doxycycline (5 mg/kg/day orally twice a day) and Rifampin (20 mg/kg/day orally twice a day) antimicrobial therapy was commenced for a total of 6 weeks (42 days) to treat brucellosis. Once starting the anti-brucellosis management, the patient did not require further FFP transfusion. The ADAMTS-13 essay result showed: extremely low ADAMTS-13 activity, which was found at 0.02 IU/ml (normal range: 0.40–1.30 IU/ml), with an antigen assay below the detection level of 0.01 IU/ml (normal range: 0.41–1.41 IU/ml); and anti-ADAMTS-13 antibodies were detected at high concentrations of >95 IU/ml (>15 IU/ml is considered positive). All these findings, along with the presentation of thrombocytopenia and hemolysis, are specific to acquired TTP.

**Figure 1:**
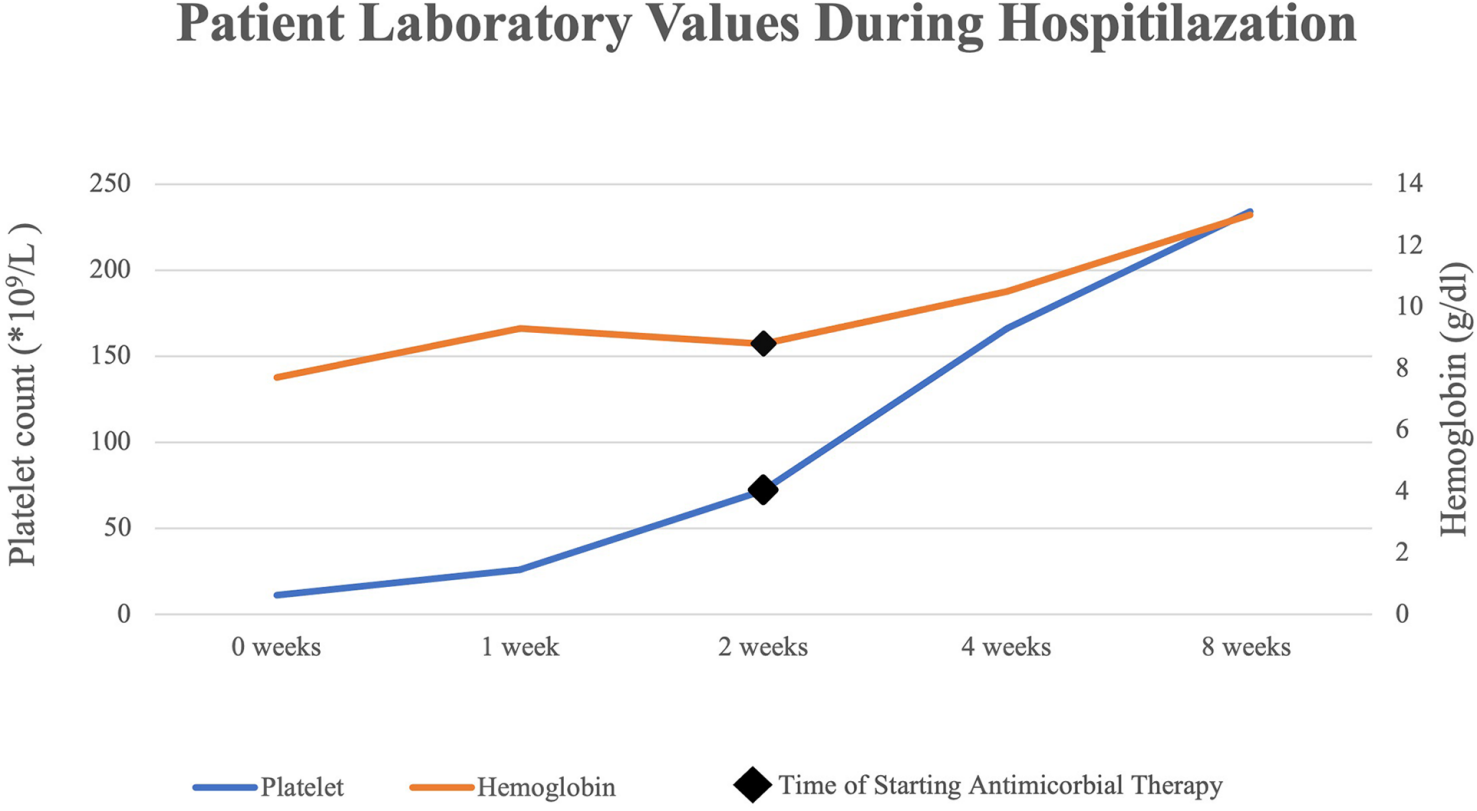
Platelet and hemoglobin levels after starting antimicrobial therapy.

Several days following the commencement of antibiotic therapy, the platelets count and hemoglobin levels recovered to the normal range (Figure 1), while the lactate dehydrogenase levels decreased substantially to a normal level.

## Discussion

The infection of brucellosis, a zoonotic disease caused by a gram-negative coccobacilli bacterium named Brucella, is considered endemic in Central Asia, the Mediterranean, and the Middle East. In most cases, its presenting symptoms are non-specific, such as fever, malaise, weight loss, and hepatosplenomegaly, making its diagnosis challenging; however, systemic involvement is not uncommon and can affect multiple systems including Central Nervous System, cardiac, Musculoskeletal, and less commonly pulmonary (1, 3).

Hematological manifestations commonly accompany this infection, mainly in the form of anemia, leukopenia & thrombocytopenia (4, 5). These hematological disturbances are attributed to hypersplenism, hemophagocytosis & bone marrow infiltration of brucella granulomas (5, 6). Fewer reported cases in the literature were linked to hemolysis. These include MAHA and, to a lesser extent, autoimmune hemolytic anemia (7). MAHA is a descriptive term for non-immune hemolytic anemia resulting from intravascular RBC fragmentation, producing schistocytes on the peripheral blood smear. TTP is one of the disorders that presents with MAHA and thrombocytopenia. It is characterized by a deficiency of von Willebrand factor (VWF) cleaving protease (ADAMTS13), which is responsible for the cleavage of ultra-large VWF multimers. When ADAMTS13 is deficient, the accumulation of such ultra-large multimers leads to microvascular thrombosis, thrombocytopenia, and hemolytic anemia (8). In addition to bleeding, its’ clinical presentation might accompany gastrointestinal, neurological, and renal involvement. The severity of this illness varies from severe life-threatening to milder vague presentation.

TTP is classified as hereditary or, more commonly, acquired. The inherited form of TTP is caused by homozygous or compound heterozygous mutations of ADAMATS 13 that are inherited as recessive traits (9). In contrast, acquired TTP is secondary to the development of inhibitory autoantibodies against ADAMTS 13. These antibodies are believed to act by neutralizing ADAMTS 13 activity or, less commonly, by enhancing its clearance (8). These antibodies were identified in almost all (94%–97%) patients with acquired TTP (10). ADAMTS13 assay and its association with TTP had been linked to multiple infectious etiologies, including influenza, malaria, particularly falciparum, HIV, and Brucella infections (11–13). Although Brucella is not a well-known infectious etiology for TTP, few reports in the literature suggest their association (Table 1) (14–21). Most of these cases were reported in adults, primarily from countries with endemic diseases, such as Turkey. The usually reported presentation was non-specific symptoms like fever, malaise, and headache, with purpuric rash being the most common sign of bleeding. Some cases have reported accompanying epistaxis, and only four cases were reported to have neurological manifestations (16–18, 21).

**Table 1:** Clinical and demographic characteristics of reported cases.

Author; year of publication	Country	Age/gender	Evidence of Brucella	Clinical features	ADAMTS-13	Treatment	Outcome
Di Mario et al. (14); 1995	Italy	19 years, male	blood culture	A, T, S	ND	Doxycycline, Rifampicin	Recovery
Yaramis et al. (15); 2001	Turkey	11 years, female	Serology, BM culture	A, T, S	ND	Doxycycline, Rifampicin	Recovery
Altuntas et al. (16); 2005	Turkey	71 years, male	Serology, blood culture	A, T, S, N	ND	Doxycycline, Rifampicin, Plasma exchange	Recovery
Erdem et al. (17); 2007	Turkey	51 years, male	Serology	A, T, S, N, R	ND	Doxycycline, TMP/SMX, Corticosteroids, FFP	Recovery
Kiki et al. (18); 2008	Turkey	19 years, female	Serology, BM culture	A, T, S, N	ND	Doxycycline, Rifampicin, Plasma exchange	Recovery
Akbayram et al. (19); 2010	Turkey	7 years, female	Serology	A, T, S	ND	Rifampicin, TMP/SMX, plasma exchange, Corticosteroids	Death
Kuperman et al. (20); 2010	Israel	27 years, female	Serology, blood culture	A, T, S	ND	Doxycycline, Gentamicin, Corticosteroids, Plasma exchange	Recovery
Bhasin A et al. (21); 2021	India	33 years, male	Serology	A, T, S, N, R	ND	Doxycycline, Rifampicin, Plasma exchange, Rituximab	Recovery
Alkhunein A et al. 2022	Saudi Arabia	9 years, male	Serology	A, T, S, R	Positive	Doxycycline, rifampicin, FFP, Corticosteroids	Recovery

N, neurological symptoms; S, schiztocytes; FFP, Fresh frozen plasma; ND, not done; A, anemia; T, thrombocytopenia; R, renal insufficiency; TMP/SMX, trimethoprim/sulfamethoxazol.

Our patient had severe epistaxis, hematemesis, and hematuria. His presentation was unique, with only two reported cases of significant epistaxis and gross hematuria (17, 21). Compared to most cases found in literature, no purpura was noted in our patient. The severity and acuity of his bleeding and hematological profile were concerning for primary hematological disease. The brucellosis diagnosis was not considered until the BMA revealed granuloma. Our patient was diagnosed with Brucella 2 weeks after admission and started on antibiotics. Our patient's presenting symptoms differ from most reported cases, as his symptoms favoured brucellosis, which led to early consideration of Brucella and administration of antibiotics within the first weeks of admission (7). The assay results obtained from our patient confirmed a low ADAMTS 13 level and the presence of antibodies, which are not previously reported in the literature to the best of our knowledge.

## Conclusion

Despite several case reports suggesting a potential association between brucellosis and TTP, our case is the first to be confirmed by ADAMTS 13 assay. Although rare, this disease manifestation is so severe that we suggest lowering the threshold for Brucella evaluation for TTP patients, particularly in areas where Brucella is prevalent. The use of the ADAMTS 13 assay in such cases should be considered when possible to confirm the diagnosis.

## Data Availability

The original contributions presented in the study are included in the article, further inquiries can be directed to the corresponding author.

## References

[1] Pappas G Papadimitriou P Akritidis N Christou L Tsianos EV. The new global map of human brucellosis. Lancet Infect Dis. (2006) 6(2):91–9. 10.1016/S1473-3099(06)70382-6 16439329

[2] Aloufi AD Memish ZA Assiri AM McNabb SJ. Trends of reported human cases of brucellosis, kingdom of Saudi Arabia, 2004–2012. J Epidemiol Glob Health. (2016) 6(1):11–8. 10.1016/j.jegh.2015.09.001 26429071 PMC7320521

[3] Al Anazi M AlFayyad I AlOtaibi R Abu-Shaheen A. Epidemiology of brucellosis in Saudi Arabia. Saudi Med J. (2019) 40(10):981. 10.15537/smj.2019.10.24027 31588475 PMC6887875

[4] Al Shaalan M Memish ZA Al Mahmoud S Alomari A Khan MY Almuneef M Brucellosis in children: clinical observations in 115 cases. Int J Infect Dis. (2002) 6(3):182–6. 10.1016/S1201-9712(02)90108-6 12718832

[5] Abdi-Liae Z Soudbakhsh A Jafari S Tomaj HE. Haematological manifestations of brucellosis. Acta Med Iran. (2007) 45(2):145–8. 10.1007/bf01739305

[6] Al-Eissa Y Al-Nasser M. Haematological manifestations of childhood brucellosis. Infection. (1993) 21(1):23–6. 10.1007/BF01739305 8449576

[7] Eskazan AE Dal MS Kaya S Dal T Ayyildiz O Soysal T. Two cases of autoimmune hemolytic anemia secondary to brucellosis: a review of hemolytic disorders in patients with brucellosis. Intern Med. (2014) 53(11):1153–8. 10.2169/internalmedicine.53.0936 24881740

[8] Verbij FC Fijnheer R Voorberg J Sorvillo N. Acquired TTP: aDAMTS13 meets the immune system. Blood Rev. (2014) 28(6):227–34. 10.1016/j.blre.2014.07.004 25213289

[9] Schneppenheim R Budde U Oyen F Angerhaus D Aumann V Drewke E von Willebrand factor cleaving protease and ADAMTS13 mutations in childhood TTP. Blood. (2003) 101(5):1845–50. 10.1182/blood-2002-08-2399 12393505

[10] Hovinga JA Lämmle B. Role of ADAMTS13 in the pathogenesis, diagnosis, and treatment of thrombotic thrombocytopenic purpura. Hematology Am Soc Hematol Educ Program. (2012) 2012(1):610–6. 10.1182/asheducation.V2012.1.610.3798654 23233642

[11] Dobson CE Tsai HM. HIV infection increases the risk of thrombotic thrombocytopenic purpura. J Thromb Circ. (2018) 4(03):127. 10.4172/2572-9462.1000127

[12] Kosugi N Tsurutani Y Isonishi A Hori Y Matsumoto M Fujimura Y. Influenza A infection triggers thrombotic thrombocytopenic purpura by producing the anti-ADAMTS13 IgG inhibitor. Intern Med. (2010) 49(7):689–93. 10.2169/internalmedicine.49.2957 20371960

[13] Kraisin S Naka I Patarapotikul J Nantakomol D Nuchnoi P Hananantachai H Association of ADAMTS13 polymorphism with cerebral malaria. Malar J. (2011) 10(1):1–8. 10.1186/1475-2875-10-366 22168261 PMC3261218

[14] Di Mario A Sica S Zini G Salutari P Leone G. Microangiopathic hemolytic anemia and severe thrombocytopenia in Brucella infection. Ann Hematol. (1995) 70(1):59–60. 10.1007/BF01715385 7827209

[15] Yaramis A Kervancioglu M Yildirim I Soker M Derman O Tas M. Severe microangiopathic hemolytic anemia and thrombocytopenia in a child with Brucella infection. Ann Hematol. (2001) 80(9):546–8. 10.1007/s002770100334 11669306

[16] Altuntas F Eser B Sarı İ Yildiz O Çetin M Ünal A. Severe thrombotic microangiopathy associated with brucellosis: successful treatment with plasmapheresis. Clin Appl Thromb Hemost. (2005) 11(1):105–8. 10.1177/107602960501100114 15678281

[17] Erdem F Kiki İ Gündoğdu M Kaya H. Thrombotic thrombocytopenic purpura in a patient with Brucella infection is highly responsive to combined plasma infusion and antimicrobial therapy. Med Princ Pract. (2007) 16(4):324–6. 10.1159/000102159 17541302

[18] Kiki I Gundogdu M Albayrak B Bilgiç Y. Thrombotic thrombocytopenic purpura associated with Brucella infection. Am J Med Sci. (2008) 335(3):230–2. 10.1097/MAJ.0b013e3180d09f19 18344698

[19] Akbayram S Dogan M Peker E Akgun C Oner AF Caksen H. Thrombotic thrombocytopenic purpura in a case of brucellosis. Clin Appl Thromb Hemost. (2011) 17(3):245–7. 10.1177/1076029609356426 20211921

[20] Kuperman AA Baidousi A Nasser M Braester A Nassar F. Microangiopathic anemia of acute brucellosis–is it a true TTP? Mediterr J Hematol Infect Dis. (2010) 2(3):4. 10.4084/mjhid.2010.031 PMC3033148 21415981

[21] Bhasin A Singal RK Chaudhary D Sharma SK Arora S Setia R Thrombotic thrombocytopenic purpura in a patient with Brucella infection. J Assoc Physicians India. (2021) 69(10):11–2. 34781663

